# The degree of altriciality and performance in a cognitive task show correlated evolution

**DOI:** 10.1371/journal.pone.0205128

**Published:** 2018-10-09

**Authors:** Pilar Chiappa, Suneeta Singh, Francisco Pellicer

**Affiliations:** 1 Departamento de Etología, Instituto Nacional de Psiquiatría Ramón de la Fuente Muñiz, Ciudad de México, México; 2 Laboratorio de Neurofisiología Integrativa, Instituto Nacional de Psiquiatría Ramón de la Fuente Muñiz, Ciudad de México, México; Mälardalen University, SWEDEN

## Abstract

Previous comparative research on the evolution of cognition has tested what we call the “altricial intelligence hypothesis”. This posits that a relationship between evolutionary changes in the altricial period length and cognition exists across animal species. However, the evidence available thus far either comes from indirect measurements of cognition or has not been conclusive. We performed a phylogenetic analysis of published data from various sources on 31 homeothermic species to test for an evolutionary association between the degree of altriciality and a direct measure of self-control. For each species, the degree of altriciality was determined based on the residual altricial period (i.e., the time from birth to fledging in birds and to weaning in mammals) on lifespan. The percentage of success in the cylinder task was the measure of self-control. Our results showed that the degree of altriciality covaried positively with the measure of self-control. Based on the results of this study, we sustain that evolutionary changes in the length of the altricial period are associated with evolutionary changes in the cognitive system used by homeotherms to perform the cylinder task.

## Introduction

Previous comparative research on how cognition has evolved largely relied on proxies for cognition. In this field, several studies have associated differences in life history strategies with differences in cognition proxies, thus identifying the developmental mode as a factor in the evolution of cognition in both birds and mammals and demonstrating that larger-brained species have extended juvenile periods and are hence in need of prolonged care [1–10]. Some authors have found an association between direct and indirect measures of cognition [11–16], and few have attempted to relate a direct measure of cognition to the developmental mode [17]. For instance, Scheiber et al. [17] predicted a link between the altricial developmental mode and social cognition. They presented an overview of studies on various aspects of the complexity of social systems in mammals and birds and arranged each species according to a dichotomous classification—altricial, precocial—of the developmental mode. Their hypothesis, however, was not supported by a qualitative analysis of social skills in several species of birds and mammals. As they suggested, a quantitative analysis is needed. Similarly, Van Schaik and colleagues recently stated that the evolution of general intelligence is concomitant with social opportunities for learning during development [18–20]. One prediction of their hypothesis is that intelligence and the frequency of opportunities for social learning during development will show a positive correlation [19]. The only comparative analysis supporting this prediction—a study of 45 solitary carnivore species [20]—found a positive correlation between the independent contrasts of the residuals of brain size in body mass and the independent contrasts of the residuals of the duration of post-weaning association with the mother in body mass. Since asocial and social learning covary across a wide range of species and depend on the same basic cognitive processes [21], this approach emphasizes not only social learning but also the important role of social circumstances during the development of cognitive traits. Nevertheless, in addition to using an indirect measure of cognition, this approach disregards the period from birth to weaning, which is also a social circumstance surrounding the development of the young and includes locomotion, thermoregulation, sensation, feeding, and cognition. Therefore, an analysis from birth based on a direct measure of cognition is still pending. Furthermore, Walker et al. evaluated the effects of group size and the percentage of dietary fruit and seeds on juvenile period length, brain size, and brain ratios while controlling for body size, lifespan, and home range in 67 species of primates. Analyzed using independent contrasts, their data suggested that differential life-history strategies come from a mix of selective social and ecological pressures of varying intensities on primate clades [22].

The latter three studies agree with what we call the “altricial intelligence hypothesis”. This posits that a relationship between evolutionary changes in altricial period length and cognition exists across animal species. To quantitatively evaluate the association between cognitive data and the degree of altriciality from birth, we used published data from various sources and carried out a comparative analysis of 31 homeothermic species.

## Methods

Few datasets have cognitive tests results generated by similar techniques in a wide variety of species. To the best of our knowledge, we selected the largest dataset that had such features. MacLean et al. [15] published the mean percentage of success in the cylinder task for 32 species (seven bird species and twenty-five mammal species, twenty of which were primates). The cylinder task measures self-control—the ability to inhibit a prepotent but ultimately counterproductive behavior when decisions are made in both social and asocial contexts [15]. Therefore, our analysis used their dataset. Following MacLean et al. [15], the arcsine transformation of the mean percentage of success in the cylinder task was used.

We defined the altricial period in birds and mammals as age at fledging (i.e., the period between hatching and flight) and age at weaning (i.e., duration of lactation), respectively. For species tested by MacLean et al. [15] in the cylinder task, we performed a semi-structured search to obtain the mean (days) of the three following variables: 1) age at fledging in birds, 2) age at weaning in mammals, and 3) lifespan in both taxa (i.e., the time from birth to death). We used three large databases to conduct our main search: Human Ageing Genomic Resources [23], Animal Diversity [24], and Pantheria [25]. We prioritized data on wild over captive conditions. When more than one value or one range was available, we calculated the mean. Our search did not produce information on some species. To fill the gaps, we used Google Scholar to search for papers published in English. The entries we used included “Latin name of the species” “fledging”; “Latin name of the species” “weaning”; “Latin name of the species” “lifespan”; and “Latin name of the species” “longevity.” We focused on the more recent publications and on those containing data on several species.

A phylogenetic tree was generated for the studied species with information from current online versions of *OneZoom Tree of Life Explorer* [26], *Timetree of Life* [27], and *10kTrees* [28]. In addition, an estimated divergence date of 15 kya was used for gray wolves and domestic dogs [29].

Life history traits are known to covary systematically across species [30]. Therefore, the evolutionary changes of one species will reflect disproportionally in another. Because altriciality is a part of lifespan, we assumed that changes in lifespan would affect the length of the former. To eliminate such effects when comparing the life history traits of species as dissimilar as the domestic dog (*Canis familiaris*) and the Western scrub jay (*Aphelocoma californica*), we worked with the phylogenetic generalized least squares (PGLS) technique [31–32], using several packages (i.e. ape [33], MASS [34], mvtnorm [35], caper [36], and nlme [37]) for the statistical software R Version 3.5.0. [38]. Lambda was estimated with maximum likelihood (ML) to calculate branch length transformations and optimize residual error structure [32]. For diagnostic purposes, we used the Plot method [39]. Following Revell’s [40] suggestion, the standardized residuals of the PGLS model of the natural logarithm of weaning or fledging on the natural logarithm of lifespan defined the degree of altriciality for subsequent analysis. Using the abovementioned software packages, PGLS was performed again to test for the predicted association between the degree of altriciality and the arcsine transformation of the mean percentage of success in the cylinder task. Once more, lambda was estimated with maximum likelihood, and the Plot Method was used for diagnostic purposes.

The slope returned by the PGLS model (λ = ML) was plotted simultaneously with the slope returned by the corresponding non-phylogenetic OLS model (with λ = 0).

## Results

Table 1 shows three sets of raw data for 31 of the 32 species tested by MacLean et al. in the cylinder task [15] (*Melospiza georgiana* is not included).

**Table 1:** Raw data and references by species.

Species	C	C ref	F/W	F/W ref	L	L ref
***Amazona amazonica*, Psittacidae (Orange-winged amazon)**	50.8	[15]	56	[41]	10950	[25]
***Aphelocoma califórnica*, Corvidae (Western scrub jay)**	76.7	[15]	20	[42]	5767	[25]
***Callithrix jacchus*, (Callitrichidae Marmoset)**	31.9	[15]	60	[43]	8322	[25]
***Canis familiaris*, Canidae (Domestic dog)**	79.1	[15]	37	[44]	8760	[45]
***Canis latrans*, Canidae (Coyote)**	95	[15]	42	[46]	7957	[25]
***Canis lupus*, Canidae (Wolf)**	77.3	[15]	35	[47]	7519	[25]
***Cebus apella*, Cebidae (Tufted Capuchin monkey)**	95.9	[15]	307	[25]	16790	[25]
***Columbia livia*, Columbidae (White carnea pigeon)**	32.5	[15]	33	[48]	12775	[25]
***Daubentonia madagascariensis*, Daubentonidae (Aye aye)**	51	[15]	197	[25]	8504.5	[49]
***Eulemur fulvus*, Lemuridae (Brown lemur)**	43.3	[15]	150	[50]	12958	[50]
***Eulemur macaco*, Lemuridae (Black lemur)**	51	[15]	165	[51]	13688	[51]
***Eulemur mongoz*, Lemuridae (Mongoose lemur)**	59	[15]	135	[52]	13213	[25]
***Eulemur rubriventer*, Lemuridae (Red-bellied lemur)**	63.8	[15]	150	[53]	7300	[25]
***Garrulus glandarius*, Corvidae (Eurasian jay)**	58.3	[15]	20	[54]	6533.5	[25]
***Gorilla gorilla*, Hominidae (Gorilla)**	94.4	[15]	1278	[55]	20075	[55]
***Lemur catta*, Lemuridae (Ring-tailed lemur)**	68.1	[15]	150	[56]	10950	[56]
***Leontopithecus chrysomelas*, Callitrichidae (Golden-headed lion tamarin)**	63	[15]	129	[57]	7774.5	[57]
***Macaca mulatta*, Cercopithecidae (Rhesus macaque)**	80	[15]	330	[58]	14600	[25]
***Melospiza melodia*, Emberizidae (Song sparrow)**	26.5	[15]	17	[59]	4124.5	[59]
***Meriones unguiculatus*, Muridae (Mongolian gerbil)**	68.9	[15]	25	[60]	730	[60]
***Pan paniscus*, Hominidae *(*Bonobo)**	95	[15]	1094	[55]	19893	[55]
***Pan troglodytes*, Hominidae (Chimpanzee)**	100	[15]	1460	[55]	21681	[55]
***Papio anubis*, Cercopithecidae (Olive baboon)**	76.3	[15]	420	[61]	9198	[61]
***Papio hamadryas*, Cercopithecidae (Hamadryas baboon)**	67.8	[15]	300	[25]	13688	[25]
***Pongo pygmaeus*, Hominidae (Bornean orangutan)**	99.1	[15]	1936	[55]	20513	[55]
***Propithecus coquereli*, Indriidae (Coquerel's sifaka)**	36.4	[15]	165	[62]	9855	[62]
***Rhinopithecuas roxellana*, Cercopithecidae (Golden snub-nosed monkey)**	35	[15]	365	[63]	9490	[63]
***Saimiri sciureus*, Cebidae (Squirrel monkey)**	33.7	[15]	177	[25]	11023	[25]
***Sciurus niger*, Sciuridae (Fox squirrel)**	66.9	[15]	56	[64]	4367.6	[25]
***Taeniopygia guttata*, Estrildidae (Zebra finch)**	52.2	[15]	21	[65]	1642.5	[65]
***Varecia variegata*, Lemuridae (Ruffed lemur)**	69.7	[15]	135	[66]	6935	[66]

From left to right, table data categories are as follows: species scientific name, species family name (species common name), mean percentage of success in the cylinder task, data reference for the cylinder task, mean age (days) at fledging in birds or at weaning in mammals, data reference for fledging/weaning, mean lifespan (days), and data reference for lifespan.

Mean percentage of success in the cylinder task ranged from 26.5 to 100 (Mean = 64.471, SD = 22.017, N = 31). Although this variable had a normal distribution (K-S = 0.107, P < 0.200, N = 31), the arcsine transformation of the percentage of success in the cylinder task was obtained.

The age at fledging in birds or at weaning in mammals ranged from 17 to 1,936 days (Mean = 305.306, SD = 472.071, N = 31). Age at weaning was more than 500 days only in the four *Hominidae* species. This variable was not normally distributed (K-S = 0.300, P < 0.001, N = 31). Normal distribution was achieved by transforming the data into their natural logarithms.

The lifespan ranged from 730 to 21,681 days (Mean = 10566.938, SD = 5328.838, N = 31). The lifespan distribution in the sample did not differ from a normal distribution (K-S = 0.111, P > 0.200, N = 31). Nonetheless, the data were transformed into their natural logarithms.

**Fig 1:**
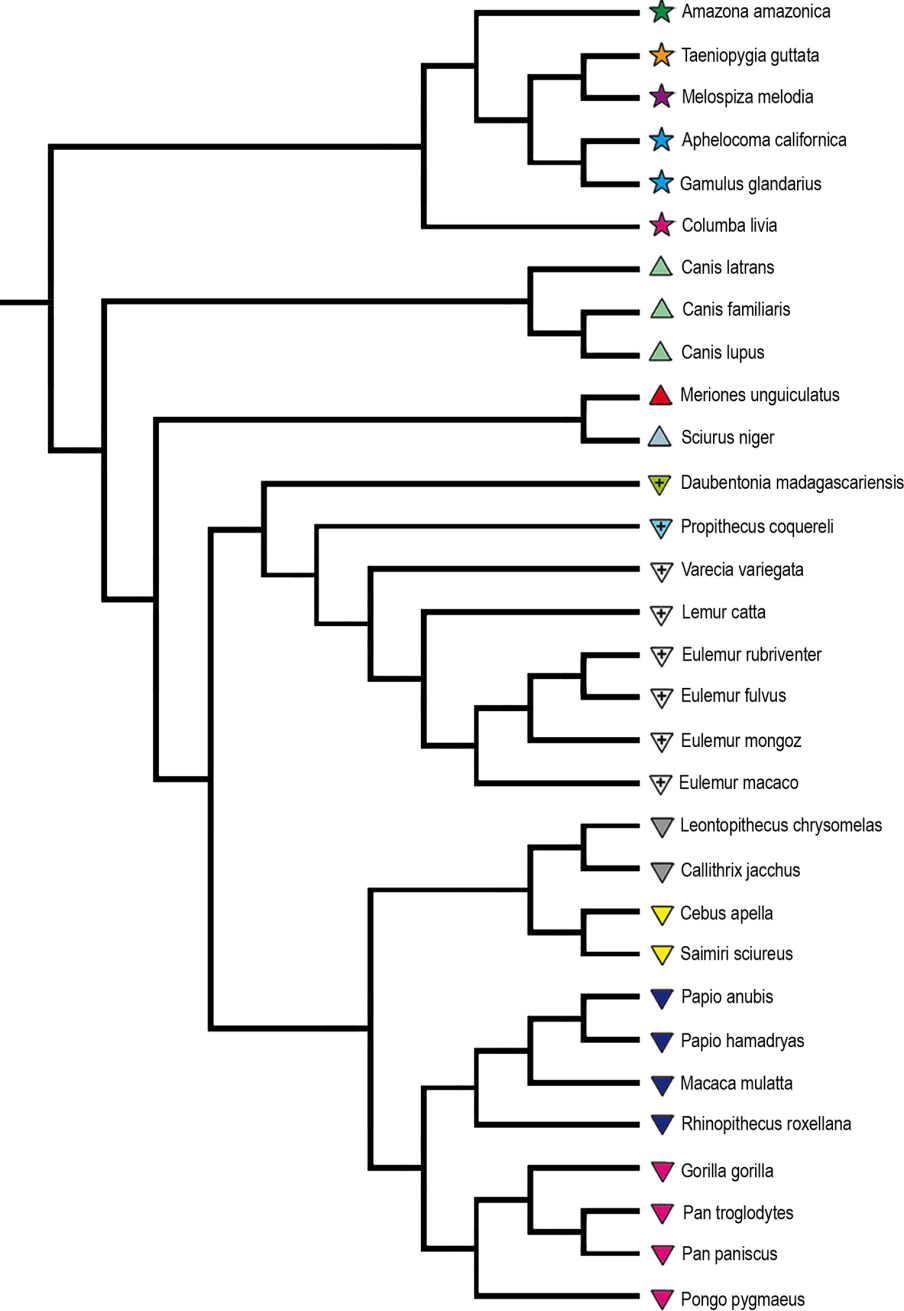
Phylogenetic tree of the 31 homeothermic species included in the analyses. Phylogenetic tree constructed with OneZoom Tree of Life Explorer [26], Timetree of Life [27], 10kTrees [28], and an estimated divergence date of 15 kya for gray wolves and domestic dogs [29]. Species names are shown on the branch tips.

In the phylogenetic tree constructed for the study (Fig 1), the parent branches were longer than the daughter branches.

PGLS model of the natural logarithm of fledging/weaning on the natural logarithm of lifespan showed a positive relationship (*r*^*2*^_*adjusted*_ = 0.183, se = 0.088, *t* = 2.781, df = 29, P < 0.010, λ = 0.987). No data showed a studentized phylogenetic residual ≥ 3.0. The distribution of the phylogenetic residuals against their expected distribution under a normal distribution fitted the line. No clear pattern emerged from the fitted values against the phylogenetic residuals. In the rest of the manuscript, the standardized residuals of this PGLS model will be called the degree of altriciality.

**Fig 2:**
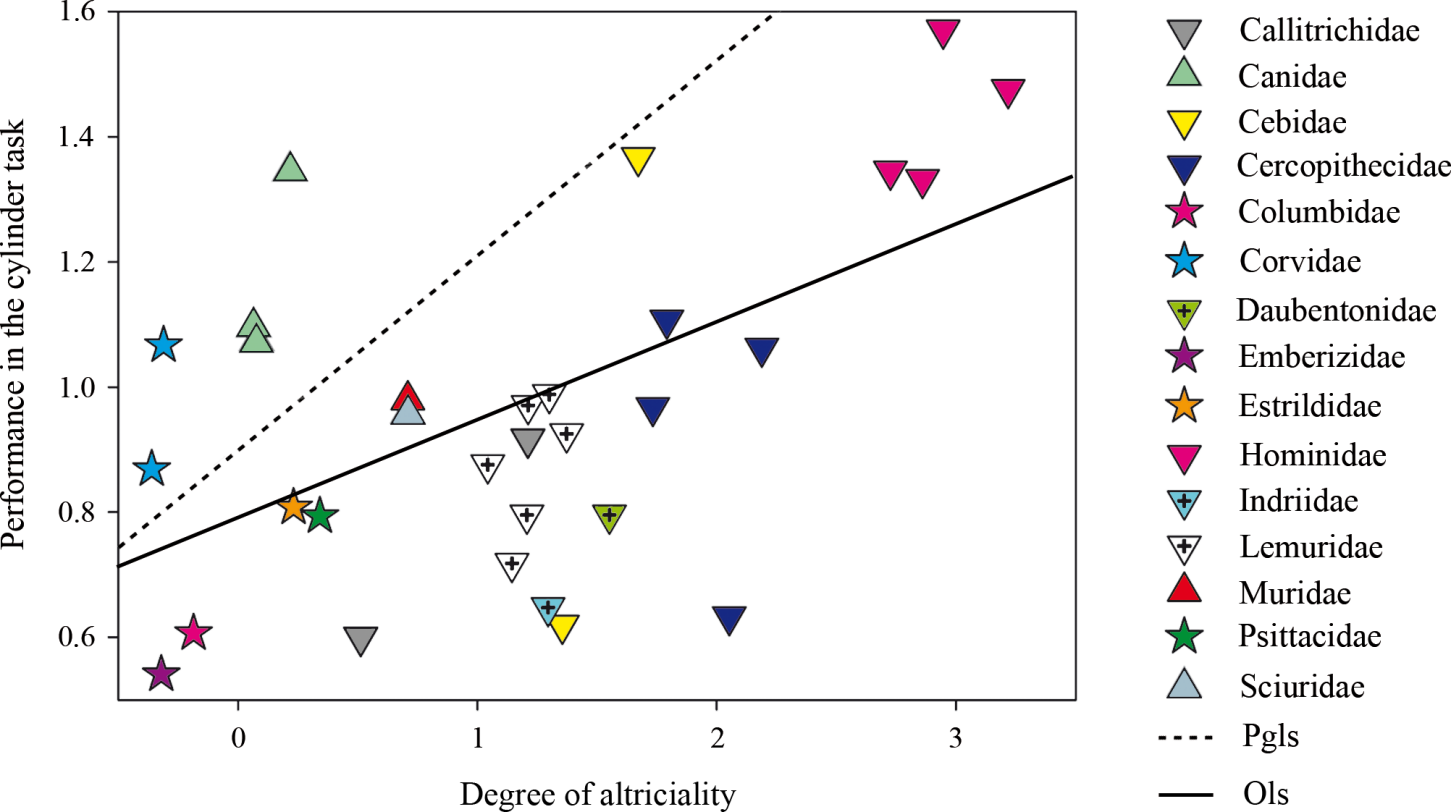
Arcsine transformation of the mean percentage of success in the cylinder task as a function of the degree of altriciality in 31 homeothermic species. The degree of altriciality was a significant predictor of the scores in the cylinder task. The straight line represents the ordinary least squares model, while the dotted line represents the phylogenetic generalized least squares model.

The PGLS model of the arcsine transformation of the mean percentage of success in the cylinder task on the degree of altriciality showed a positive regression (*r*^*2*^_*adjusted*_ = 0.2409, se = 0.0905, *t* = 3.2433, df = 29, P < 0.003, λ = 0.94) (Fig 2). No data showed a studentized phylogenetic residual ≥ 3.0. The distribution of the phylogenetic residuals against their expected distribution under a normal distribution fitted the line. No clear pattern emerged from the fitted values against the phylogenetic residuals.

The corresponding OLS model (i.e., λ = 0) returned a positive regression of the arcsine transformation of the mean percentage of success in the cylinder task on the degree of altriciality (*r*^2^_*adjusted*_ = 0.2464, se = 0.0434, *t* = 3.2880, df = 29, P < 0.003).

## Discussion

Based on the results of the phylogenetic general least squares, we can postulate that the degree of altriciality is associated with the percentage of success in the cylinder task. Of the species included in the analysis, some outperformed others in the cognitive test. The offspring of the outperformers had longer rearing periods and therefore greater degrees of altriciality than did the offspring of the other species. As predicted by the altricial intelligence hypothesis, this finding suggests that such traits are evolutionarily related.

The relationship between the relative age at weaning and relative brain growth [67] suggests that brain development is accompanied by an altricial period. This is in line with our results.

The association between a measure of self-control and the degree of altriciality was obtained by accounting for the phylogenetic relationships among species. As with any other phylogenetic analysis, this result relies heavily on what species are included or excluded. Although the dataset that we analyzed included a few groups of closely related species (e.g., the three species of the order *Carnivora*), the sample consisted mainly of distantly related species (e.g., the two species of the order *Rodentia*). Furthermore, the dataset included species known for their cognitive performance. A further analysis of a sample of species of high cognitive reputation and their phylogenetic sister taxa could reveal changes in the strength of this association.

The only previous study of the relationship between cognition and time of exposure to social circumstances in offspring used an indirect measurement of cognitive capacity and was performed in carnivore species only [20]. Our findings are based on previously published data from homogeneous cognitive tests carried out in homeothermic species (birds and mammals). We could have increased our sample size by including, for instance, the four bird species subjected to the cylinder task by Kabadayi et al. [68]. Rather, we chose to use a homogeneous dataset.

Previous attempts to study the relationship between the developmental mode and cognitive complexity have yielded varying results. For example, Scheiber et al. hypothesized that social complexity is associated with the developmental mode (i.e., altricial, precocial) [17]. Their hypothesis, however, was not supported by a qualitative analysis of social skills in several species of birds and mammals. It is possible that the differences between their results and ours are due to the types of variables used in each study. A dichotomous classification of the developmental mode may overlook minor differences between species, while the length of a developmental stage may reflect more variations among species in the altricial-precocial spectrum. The degree of altriciality is likely to be positively associated with one of the indicators of social complexity considered by Scheiber et al.—for instance, the percentage of aggressive coalition [17]. Furthermore, the degree of altriciality may be used to understand the relationship between a relatively enlarged telencephalon and the developmental mode, an assumption made by Charvet and Striedter [69]. For example, *Anser anser* is a precocial duck species in terms of the readiness for feeding hatchlings [1]. Fledging occurs 55 days after birth, and the ducks´ lifespan is 7,300 days [70]. The phylogenetic residual of the length of the species altricial period on lifespan is expected to be similar to that of the marmoset and of the domestic dog. This may account for the ducks´ having a large telencephalon despite being precocial in other developmental aspects, including post-hatching brain growth [69].

Starck and Ricklefs used the functional capacity of tissue to describe the developmental state of a neonate [1]. With this metric, it is possible to investigate species from different taxonomic groups. Like theirs, our metric allowed the study of both birds and mammals. However, their metric did not show a phylogenetic pattern among dozens of bird species and was uncorrelated with either their phylogeny or their brain mass relative to body mass. Consequently, the authors argued for the need to 1) identify the ecological pressures that promote changes in developmental mode and 2) focus on other developmental aspects that might correlate with brain size. Their second suggestion confirms our approach because the degree of altriciality depends on social aspects. Although lactation and regurgitation satisfy primarily energetic needs during the altricial period, they are concomitant with other aspects of altricial dependency, such as cognitive development.

It would have been interesting to compare our degree of altriciality with other developmental mode measurements, as did Starck and Ricklefs with their metric [1]. However, placing the species included in our analysis within the altricial-precocial spectrum according to classical categories would have yielded minimal variation. A recent evolutionary analysis of the developmental modes in birds [71] revealed that all of the birds in our sample could have been classified as altricial or super-altricial.

The degree of altriciality represents a phylogenetic account of the altricial period (i.e., age at fledging/weaning) relative to lifespan. Research has suggested that lifespan itself may be an allometric consequence of other characteristics subjected to selective pressures [72]. The maximum lifespan in homeothermic species varies greatly with body mass (i.e., [72, 73]). A longer lifespan is likely to promote larger bodies. Some authors have argued that lifespan itself is a target of selective processes because it shows considerable variation and is heritable (i.e., [74]). Both age at fledging and age at weaning are points in the lifespan. Therefore, they can be used to obtain a scale of altriciality.

Ghirlanda et al. developed a mathematical model to study the coevolution of behavioral repertoire and intelligence under selection pressure for efficient learning of functional sequences of behavior. They noted that learning time (as measured by lifespan) is essential for the acquisition of intelligent behavior [75]. Our results are in line with this interpretation. Similarly, Walker et al. found an association between the end of the growth period and the nonvisual neocortex ratio in primates [22]. This result underlines the evolutionary importance of the length of juvenile periods in indirect measurements of cognition. Our results reinforce this interpretation. In addition, their data showed no evidence for a single initiator of slow life history strategies. In fact, they found that lifespan is an important determinant of brain size in New World monkeys, as is home range in Old World monkeys [22]. MacLean et al. [15] showed that absolute endocranial volume covaried positively with the very same cognitive data that we used here. Based on these findings, it would be interesting to investigate whether the degree of altriciality and any indirect measurement of cognition (brain volume, ratio of frontal cortex to rest of cortex, etc.) in homeothermic animals show an association that is similar to the one we presented here.

## Conclusion

Based on the results of the phylogenetic general least squares, we can conclude that the degree of altriciality is associated with the percentage of success in the cylinder task, which was predicted by the altricial intelligence hypothesis.
